# Supplementary material for: Comparison of the Patency and Regenerative Potential of Biodegradable Vascular Prostheses of Different Polymer Compositions in an Ovine Model
Source: Int J Mol Sci. 2023 May 10;24(10):8540. doi: 10.3390/ijms24108540 (PMC10218148; doi:10.3390/ijms24108540)
Supplement: Supplementary file 1 [file ijms-24-08540-s001.zip › ijms-2309726-supplementary.pdf]

Supplementary Figure S1

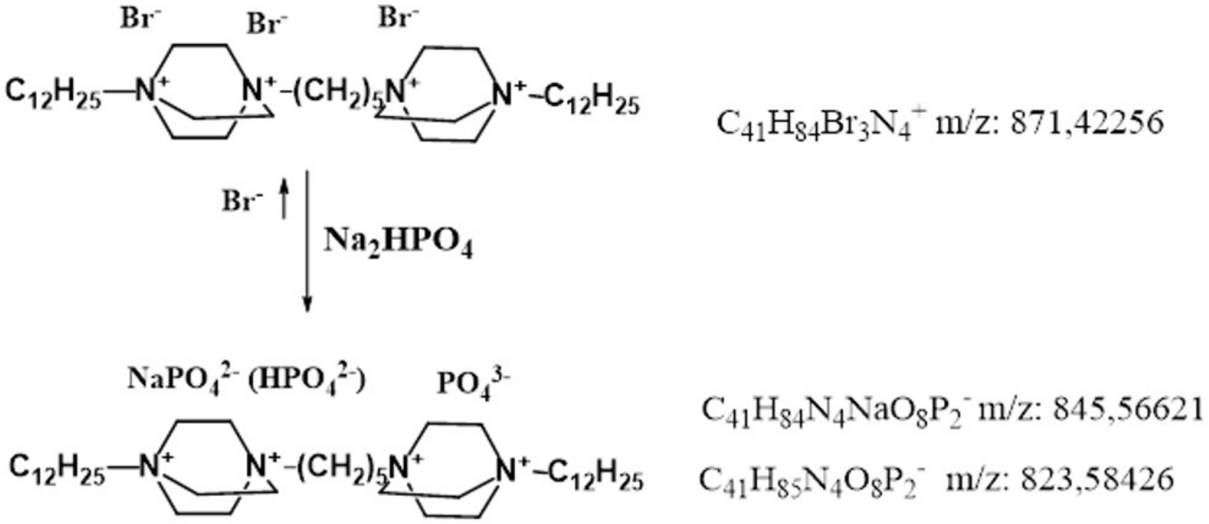


**Supplementary Figure S1.** Chemical structure of cationic amphiphile 1,5-bis-(4-tetradecyl-1,4-diazoniabicyclo[2.2.2]octan-1-yl)pentane tetrabromide after incubation in phosphate-buffered saline.

Supplementary Figure S2

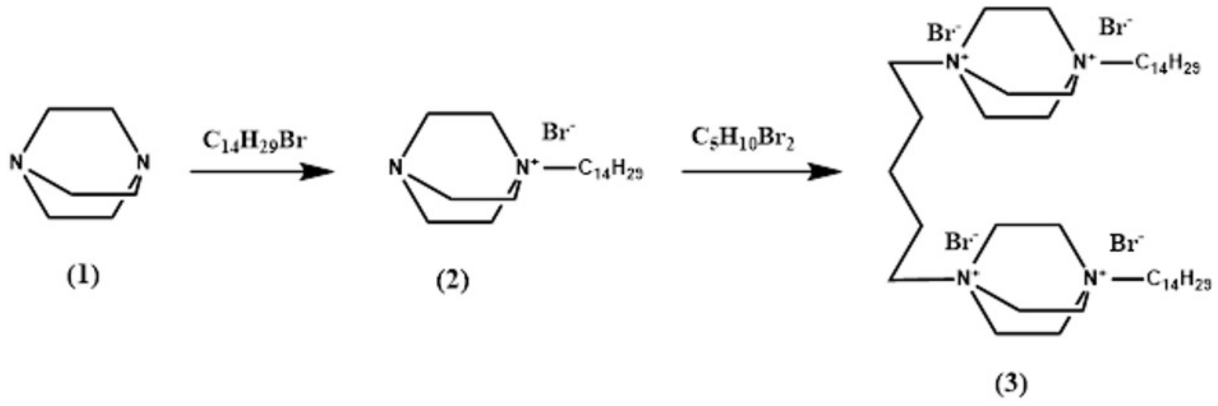


**Supplementary Figure S2.** Scheme of the synthesis of 1,5-bis-(4-tetradecyl-1,4-diazoniabicyclo[2.2.2]octan-1-yl)pentane tetrabromide.

Supplementary Figure S3

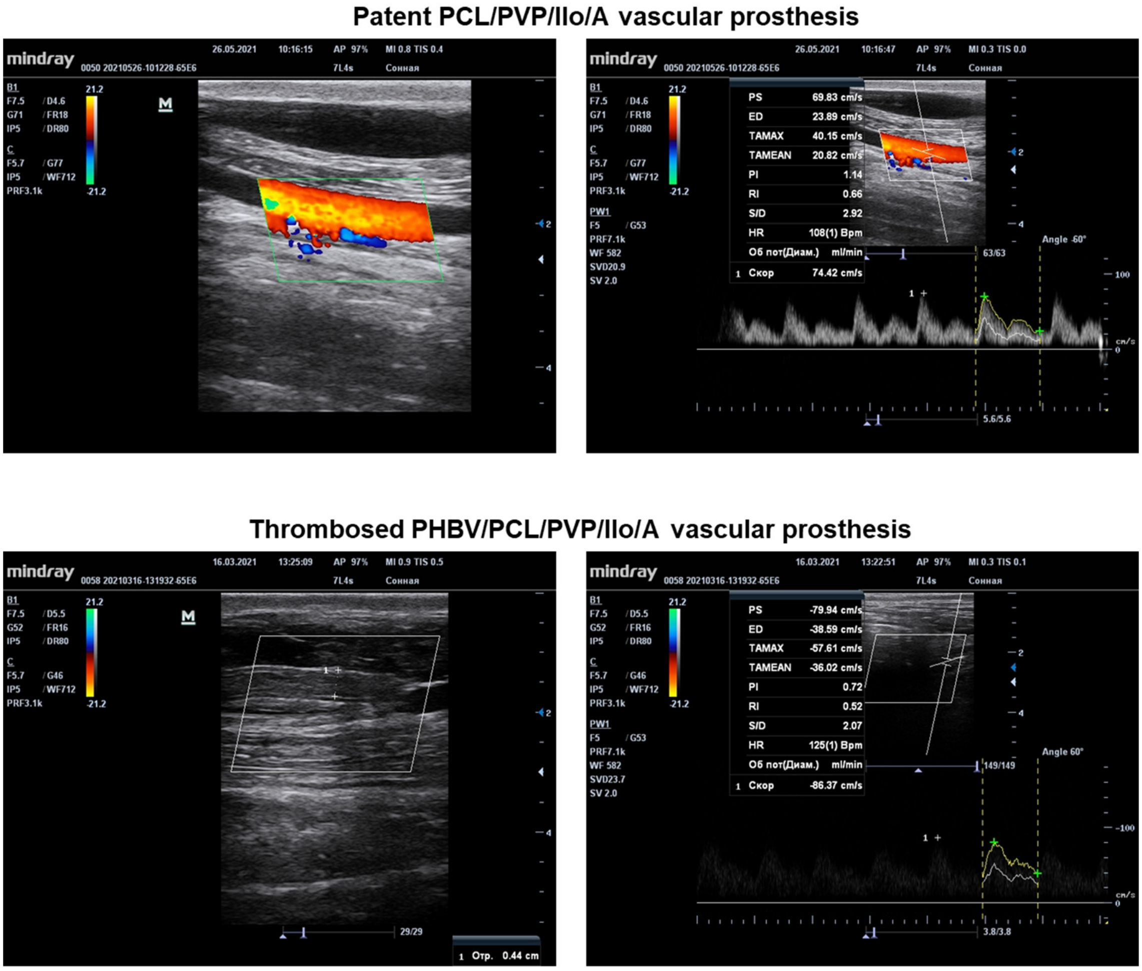


**Supplementary Figure S3.** Representative ultrasound Doppler images of patent PCL/PVP/Ilo/A and thrombosed PHBV/PCL/PVP/Ilo/A vascular prostheses into the ovine carotid artery 1 day after sugery.

Supplementary Figure S4

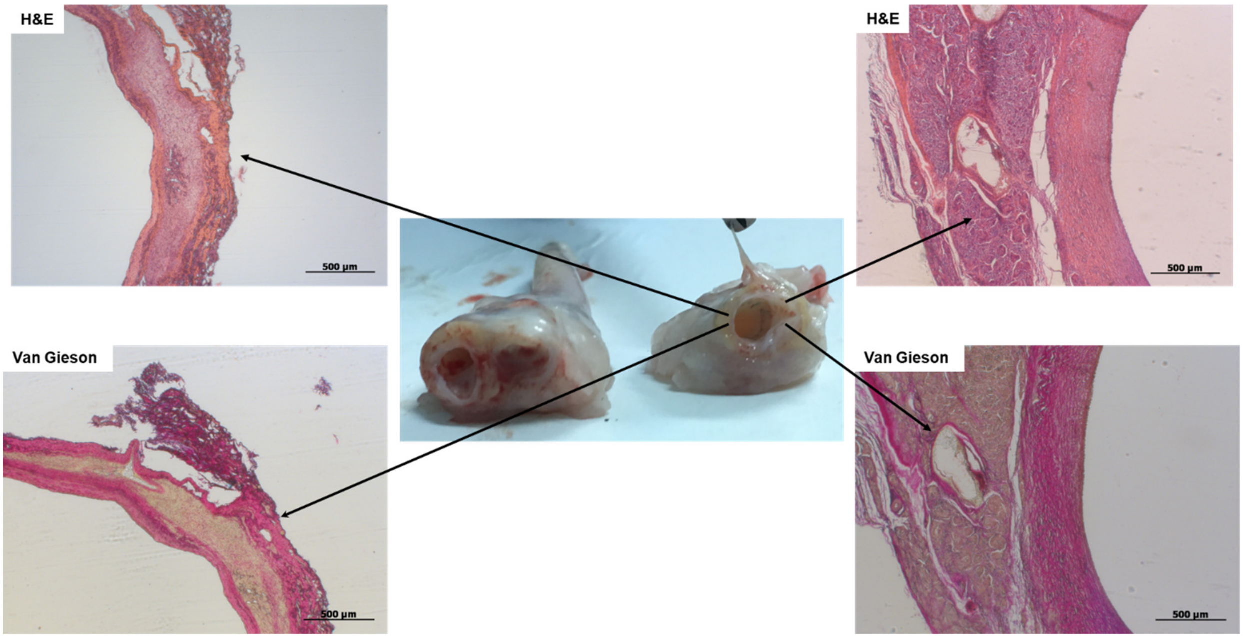


**Supplementary Figure S4. Morphological observation of the PCL/PVP/Ilo/A prostheses after 6-month implantation in the ovine carotid artery: histological examination of different cross-section areas using hematoxylin and eosin (H&E) and Van Gieson staining, with a scale bar of 500 μm.**
